# Functional shifts of the dual-substrate phosphoribosyl isomerase PriA from primary to specialized metabolism in rare Actinomycetota

**DOI:** 10.1099/mgen.0.001634

**Published:** 2026-04-07

**Authors:** Luis Rodrigo Rosas-Becerra, Andrés Arredondo-Cruz, Nelly Sélem-Mojica, Francisco Barona-Gómez

**Affiliations:** 1 Evolution of Metabolic Diversity Laboratory. Centro de Investigación y de Estudios Avanzados del Instituto Politécnico Nacional (Cinvestav-IPN), Irapuato, Guanajuato, México; 2 Institute of Biology Leiden, Leiden University, Leiden, The Netherlands; 3 Centro de Ciencias Matemáticas, UNAM, Morelia, Michoacán, Mexico

**Keywords:** *Actinomycetota*, adechlorin, biosynthetic gene cluster, enzyme evolution, gene expansion, genome mining, PriA

## Abstract

Evolutionary functional innovations can occur while gene families expand and two homologous (or analogous) genes co-occur. PriA is a dual-substrate enzyme family exclusive to *Actinomycetota*, with activities in l-histidine and l-tryptophan biosynthesis (HisA and TrpF activities) that evolved after the loss of the *trpF* gene. Since this gene loss, PriA has undergone multiple functional gain and loss events in central metabolism. Here, we report further evolutionary scenarios of PriA. First, in *Ornithimicrobiaceae*, a rare *Actinomycetota* family, PriA coexists with HisA, concomitant with the loss of its HisA but not TrpF activity, and is recruited in a physiological genome context. Second, a *priA* homologue, *adeK*, is located in the biosynthetic gene cluster of the specialized metabolites adechlorin and 2′-amino-2′-deoxyadenosine. The *adeK* gene is encoded in several genomes of the *Streptosporangiaceae* family and coexists with the expected and conserved *priA*. In this scenario, the PriA enzymes conserve both activities, whereas AdeK has only detectable levels of HisA activity, which might be related to specificity for a chlorinated intermediary during adechlorin biosynthesis. The *adeK* gene was first identified in strain ATCC-39365, previously designated as *Actinomadura* sp., but reclassified here as *Nonomuraea* sp. We further identified unprecedented adechlorin/2′-amino-2′-deoxyadenosine/pentostatin producers within the *Microbispora* and *Streptosporangium* genera. Phylogenomic analysis revealed additional recruitments of *priA* and *hisG* homologues into diverse uncharacterized biosynthetic gene clusters taxonomically related to the nucleoside antibiotics adechlorin, pentostatin and pyrazomycin. These findings highlight how enzyme family expansions and recruitment drive metabolic functional innovation in *Actinomycetota*, involving both gene loss and gain throughout central and specialized metabolism, and how evolutionary genome mining allows unbiased natural product discovery.

**Impact Statement**

Gene family expansion within enzyme families is a crucial evolutionary driver of metabolic innovation and biochemical diversity. In this study, we investigated the evolutionary dynamics of the dual-substrate enzyme family PriA across the *Actinomycetota* phylum and uncovered two unexpected scenarios of functional shift: (1) in *Ornithimicrobiaceae*, PriA coexists with HisA but retains only TrpF activity; and (2) in *Streptosporangiaceae*, a PriA homolog, AdeK, conserves only HisA activity, and its encoding gene is recruited into biosynthetic gene clusters (BGCs) responsible for the two independent pathways of the nucleoside antibiotics adechlorin and 2′-amino-2′-deoxyadenosine (2′-amino dA). Notably, the identification of genomic vicinities of *adeK* homologues revealed both known and uncharacterized biosynthetic gene clusters associated with nucleoside antibiotic biosynthesis across diverse taxa. These findings provide new insights into the interplay between central and specialized metabolism and underscore how enzyme family expansions and gene recruitment into BGCs can drive biochemical innovations. Our work demonstrates the utility of evolutionary genome mining to uncover hidden biosynthetic potential in rare microbial lineages.

## Data Summary

The following external software was used for this research:

- EvoMining DOI: 10.1099/mgen.0.000260. Available from https://github.com/nselem/evomining
- CORASON DOI: 10.1038 /s41589-019-0400-9. Available from https://bigscape-corason.secondarymetabolites.org
- blastp. Available from: https://blast.ncbi.nlm.nih.gov/Blast.cgi
- Flye DOI: 10.1038 /s41587-019-0072-8. Available from: https://github.com/mikolmogorov/Flye
- Databases have been deposited at Zenodo; DOI: 10.5281/zenodo.15968994 https://zenodo.org/records/15968994

Trees and metadata have been deposited in Microreact: PriA in *Actinomycetota* (https://microreact.org/project/m8EnV8tU28h5ARpoY4nxmY-priarerooted031224) and PriA in *Ornithinimicrobiaceae* and *Streptosporangiaceae* (https://microreact.org/project/8KvmkNd7VSo2ARcTkH3rkr-ornistreppria091224).

The genome sequence of *Nonomuaraea* (*Actinomadura*) sp. ATCC-39365 was deposited in the Assembly database of the National Center for Biotechnology Information with the accession number: CP184335.

All supporting data and protocols have been provided within the article or through supplementary data files.

## Introduction

Diversification of metabolic functions is a key evolutionary driver that enables microorganisms to adapt to complex ecological niches and resource limitations. Central to this process are gene expansions, which often serve as raw materials for subfunctionalization and neofunctionalization [1,2]. The expansion of gene families associated with central metabolism facilitates the development of novel catalytic functions and substrate specificities, thereby enabling the evolution of specialized metabolism from central pathways [3,5].

The phylum *Actinomycetota* is a diverse group of Gram-positive bacteria that inhabit a wide range of ecological niches [6]. It is distinguished by its remarkable metabolic diversity, including numerous primary metabolic pathways and specialized biosynthetic capacities for natural products [7,8]. Phylogenetic and phylogenomic studies in *Actinomycetota* have explored the interplay between genome dynamics, the evolution of metabolic pathways and the functional diversification of enzymes in both central metabolism [9] and specialized metabolism [10]. The latter often involves the recruitment of enzymes into biosynthetic gene clusters (BGCs), which are discrete genomic regions comprising colocalized genes that collectively encode the enzymatic machinery required for the synthesis of specialized metabolites [11].

**Fig. 1.**
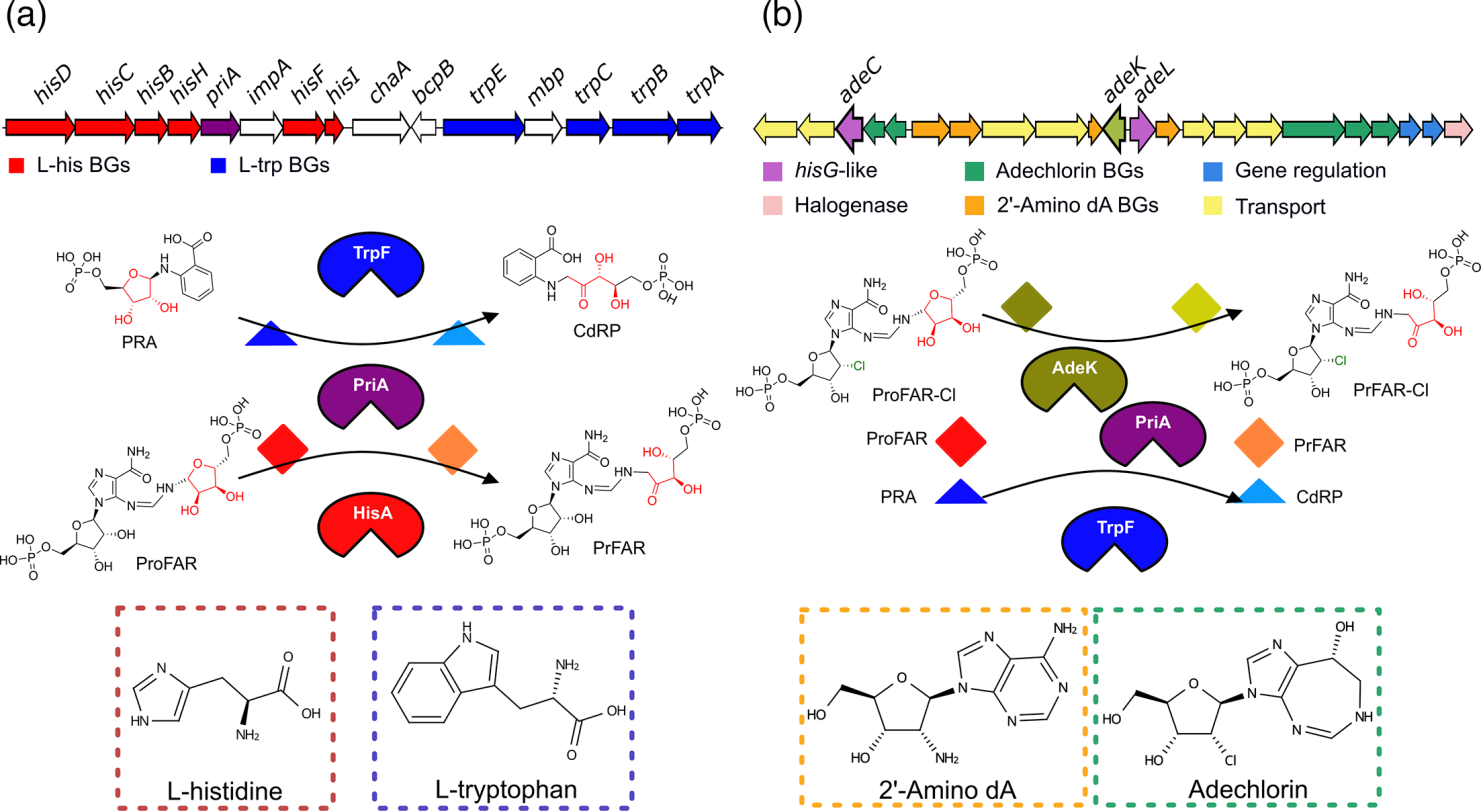
(*βα*)_8_ isomerases PriA and AdeK. (**a**) Isomerization reactions in l-histidine and l-tryptophan are catalysed by PriA or phosphoribosyl isomerase A at the same catalytic site in *Actinomycetota*. The same reactions are independently catalysed by HisA or ProFAR isomerase and TrpF or PRA isomerase in most bacteria. The *priA* gene is in a genomic context that involves *his* and *trp* genes for the synthesis of l-histidine and l-tryptophan, respectively. (**b**) *adeK* is a *priA* homologue encoded in the *ade* BGC of adechlorin and 2′-amino dA from strain ATCC-39365. AdeK catalyses the isomerization of ProFAR to PRFAR (shown in this study). The proposed isomerization of ProFAR-Cl into PRFAR-Cl is a hypothetical step derived from the adechlorin biosynthetic pathway, which begins with the chlorinated substrate 2′-chloro-2′-deoxyadenosine monophosphate and reflects the sub-specialization of AdeK in ProFAR isomerase activity.

The phosphoribosyl isomerase A (PriA) enzyme family is specific to *Actinomycetota* and exemplifies an evolutionary adaptation with dual functionality. PriA functions as a dual-substrate (*βα*)₈-barrel phosphoribosyl isomerase, which participates in both l-histidine and l-tryptophan biosynthesis pathways, performing roles analogous to those of HisA and TrpF, respectively [12]. HisA and TrpF catalyse mechanistically similar isomerization reactions. HisA converts N-[(5-phosphoribosyl)formimino]-5-aminoimidazole-4-carboxamide ribonucleotide (ProFAR) into N-[(5-phosphoribulosyl)formimino]-5-aminoimidazole-4-carboxamide ribonucleotide (PRFAR), whereas TrpF transforms N-(5′-phosphoribosyl)-anthranilate (PRA) into 1-(O-carboxyphenylamino)-1′-deoxyribulose-5′-phosphate (CdRP) (Fig. 1a) [13,16]. Notably, in the phylum *Actinomycetota*, *trpF* is absent in most genomes, and PriA compensates for this loss by catalysing both reactions (Fig. 1a) [12]. The dual substrate affinity of PriA makes it a suitable enzyme model for studying the evolutionary dynamics of the loss and gain of functions under various metabolic scenarios. In *Actinomadura* sp. ATCC-39365, the recruitment of PriA in specialized metabolism has been previously identified [17]. This *priA* homologue, designated as *adeK,* is situated within the *adeEDCBAFGHIJKLMNOPQRSTV* BGC (*ade* BGC), which encodes the synthesis of adechlorin and 2′-amino dA (Fig. 1b) [17,18].

**Fig. 2.**
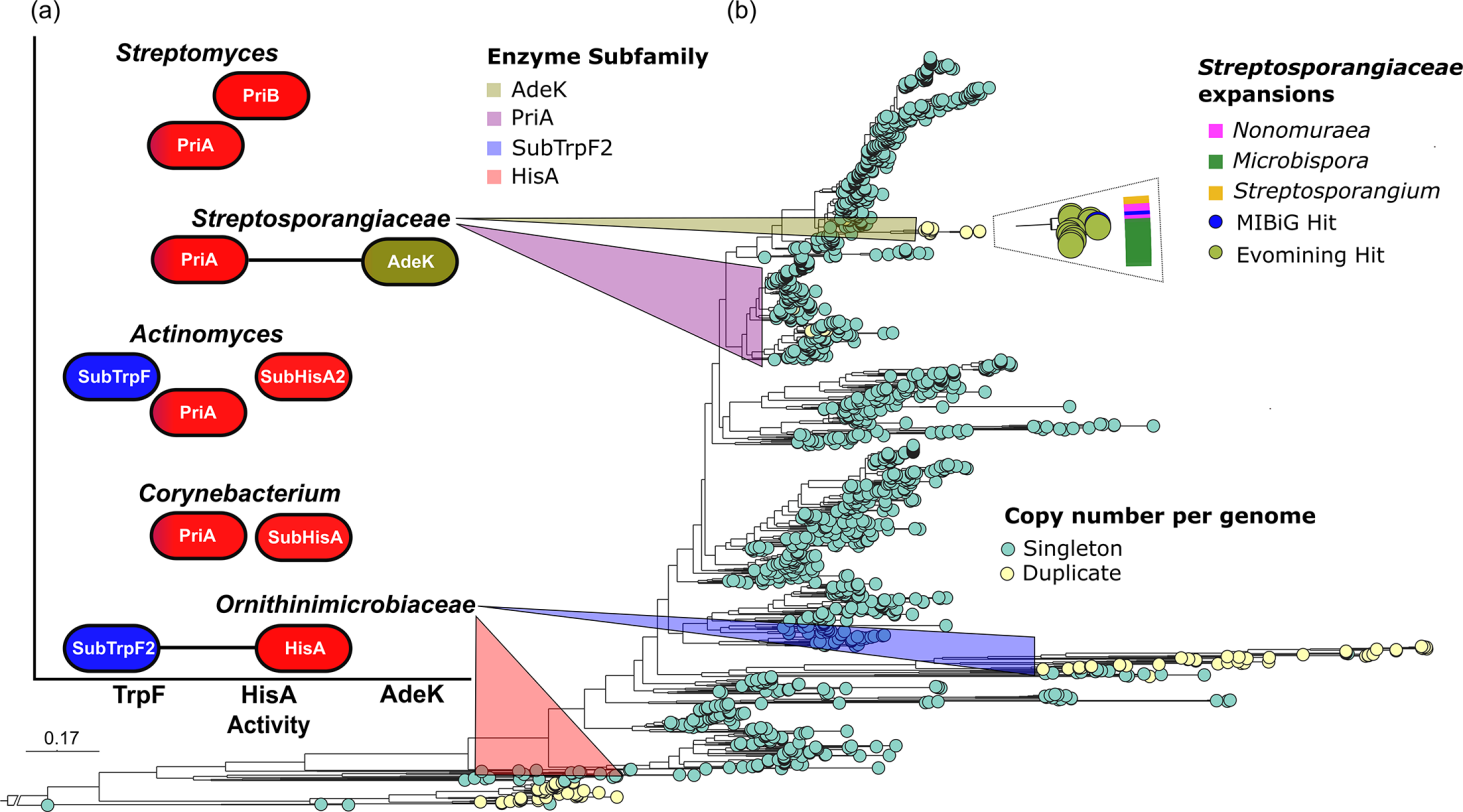
The PriA family exhibits expansions and functional shifts in *Actinomycetota*. (**a**) PriA enzyme family displays functional versatility across *Actinomycetota* lineages, with the subfamilies TrpF (blue), HisA (red) and AdeK (green) showing distinct catalytic activities. PriA demonstrates dual-activity TrpF and HisA (blue and red), whereas AdeK shows HisA activity (red) and HisA-like specialized activity (green). Black lines indicate homologue co-occurrence. (**b**) PriA phylogeny of the *Actinomycetota* phylum shows expansions in *Ornithinimicrobiaceae* and *Streptosporangiaceae*, marked with coloured shades over the phylogeny. Homologues of HisA and PriA (SubTrpF2) co-occur in *Ornithinimicrobiaceae*. In *Streptosporangiaceae*, AdeK represents a PriA expansion recruited into specialized metabolism within three genera: *Nonomuraea*, *Microbispora* and *Streptosporangium*.

In central metabolism, functional adaptations of PriA in response to genome dynamics have led to the emergence of the SubHisA, SubHisA2, SubTrpF and PriB subfamilies (Fig. 2a), which exhibit distinct substrate specificities [19,21]. These subfamilies were identified through phylogenetic analyses in *Corynebacterium* (SubHisA), *Actinomycetaceae* (SubHisA2 and SubTrpF) and *Streptomyces* (PriB), in which horizontal gene transfer, gene loss and co-occurrence of functionally analogous *trpF* have been the evolutionary driving forces shaping such specializations. Although such functional shifts or subfunctionalization have occurred in single-copy *priA* contexts, metabolic scenarios in which gene expansions within the PriA enzyme family act as the driving force have remained elusive.

EvoMining, an evolutionary genome-mining approach that recapitulates enzyme expansion events, has enabled the identification of expanded, repurposed enzyme families with the potential to catalyse new conversions in natural product biosynthesis [4,10]. Here, we used EvoMining to analyse the *priA* gene family across 1,541 *Actinomycetota* genomes and uncovered two distinct evolutionary scenarios: (i) co-occurrence of *hisA* and *priA* homologues in *Ornithinimicrobiaceae*, where PriA subfunctionalized to TrpF activity (SubTrpF2) and is recruited in an uncharacterized physiological genome context, and (ii) recruitment of the *priA* homologue *adeK* into specialized metabolism in *Streptosporangiaceae*, where AdeK retained only HisA activity. *adeK* was previously annotated in the *ade* BGC of *Actinomadura* sp. ATCC-39365 (here reassigned as *Nonomuraea* sp.) [17,18]. Our phylogenomics analyses revealed that *adeK* is consistently recruited into *ade* BGCs across *Nonomuraea*, *Microbispora* and *Streptosporangium* strains, all of which are confirmed as producers of adechlorin, 2′-amino-dA and pentostatin. Broader comparative analyses further showed the recruitment of *priA* and *hisG* homologues into uncharacterized BGCs evolutionarily related to the nucleoside antibiotics adechlorin, pentostatin and pyrazomycin. Altogether, our findings (i) highlight distinct evolutionary trajectories of PriA expansions and recruitment in central and specialized metabolism within *Actinomycetota*, (ii) reveal a broad recruitment strategy of related gene expansions in nucleoside antibiotic biosynthesis and (iii) underscore the power of evolutionary genome mining to uncover non-canonical BGCs with potential for drug discovery.

## Methods

### Evolutionary genome mining using EvoMining

The EvoMining algorithm [4] was employed to identify significant expansions of the PriA protein family across *Actinomycetota*. EvoMining requires three core databases: the Genome database (DB), Enzyme DB and Natural Product (NP) DB. The Enzyme DB contains seed sequences for the PriA family, including those in the strain ATCC-39365, *Microbispora fusca* DSM 104648 and *Streptosporangium becharense* DSM 46887, as well as a HisA sequence from *Acidimicrobium ferrooxidans*. To search expansions, two Genome DBs were used: (1) a focused database comprising representative genomes from *Ornithinimicrobiaceae* and *Streptosporangiaceae* and (2) a broader *Actinomycetota* Genome DB, consisting of 1,284 genomes from a previously curated dataset [4], supplemented with the aforementioned *Ornithinimicrobiaceae* and *Streptosporangiaceae* genomes. The NP DB included all amino acid sequences from the MIBiG v3.0 repository [22].

EvoMining performs blastp searches (*E*-value ≤0.001, bitscore ≥100) using Enzyme DB sequences as queries against the Genome DB to detect expanded enzyme families. Significant expansions are defined as enzyme families with at least one genome containing copy numbers exceeding the mean plus two standard deviations. Expanded enzyme families are queried against the NP DB to identify recruited homologues into specialized metabolism. EvoMining classifies enzyme copies into three categories: (i) conserved (central metabolism; bidirectional best hits to Enzyme DB), (ii) natural product-biosynthesis recruited (matches to NP DB) and (iii) novel or uncharacterized extra copies.

Enzyme family sequences were aligned with muscle v3.2 and curated using Gblocks v0.91b (minimum block length: 5, maximum non-conserved positions: 10, gaps allowed in <50% of sequences). EvoMining phylogenies were inferred using FastTree v2.1 (approximately maximum likelihood), and trees were colour-labelled using Newick Utilities to indicate predicted metabolic fates: red (conserved metabolism), blue (MIBiG hits), green (novel EvoMining predictions), cyan (antiSMASH-predicted BGCs), purple (transitions into conserved and specialized metabolites) and grey (unknown fate). All these actions are systematized in the EvoMining algorithm. Trees and associated metadata, such as copy number and functional annotation, were visualized using Microreact [23].

### Genome curation and database integration

From the National Center for Biotechnology Information (NCBI) repository database, 588 genome sequences were retrieved, comprising all genomes available as of August 2024, classified under the *Streptosporangiaceae or Ornithinimicrobiaceae* families. *A. ferrooxidans* was included as an outgroup. All genomes were uploaded to the Rapid Annotation using Subsystems Technology (RAST)[24] server for functional gene annotation. The average gene content per contig was calculated by dividing the ratio between the genome size in base pairs by 1,000 bp and the number of contigs. Genomes were filtered, keeping only those with more than ten genes on average per contig. Through manual curation of genomic sequences, duplicated genomes were identified and removed as they would have led to erroneous expansion signals in the analysis. Subsequently, the *Ornithinimicrobiaceae* and *Streptosporangiaceae* genomic database was integrated with 257 genomes.

### Genome context visualization using CORASON

We employed CORASON [25] to identify, visualize and explore the evolutionary relationships among BGCs. CORASON requires three inputs: a custom genomic database, a reference BGC and a query protein within the reference cluster. The genomic databases used were the same as those described above for EvoMining. Depending on the analysis, different query sequences and reference clusters were selected, including HisA and PriA from *Ornithinimicrobium murale* and PriA and AdeK from *Nonomuraea* (*Actinomadura*) sp. ATCC-39365. These genomes were flagged to indicate the presence of the reference BGC in each respective analysis.

CORASON performs blastp searches (*E*-value ≤0.001) to identify homologues of the query gene across the genomic database. For each hit, a genomic region spanning ten genes upstream and downstream is extracted to form a temporary database. Within these regions, CORASON searches for additional homologues of the reference BGC using blastp. Clusters containing at least two reference homologues, including the query gene, are retained to build the Cluster Variation Database (CVD).

To reconstruct BGC phylogenies, CORASON defines a conserved core composed of gene families that are multidirectionally best hits across all clusters in the CVD. These core sequences are concatenated, aligned with muscle v3.8.31, and curated using Gblocks to remove poorly aligned regions. Phylogenetic trees are inferred using FastTree v2.1.10. BGCs are visualized and aligned relative to the query gene, with gene similarity displayed via a colour gradient.

### Testing for relaxed selection

We used RELAX [26] to assess shifts in selective pressure on the AdeK branch (test) compared to the PriA branch (reference) from the *Streptosporangiaceae* PriA phylogeny built by EvoMining. RELAX is a phylogenetic method designed to detect shifts in the intensity of natural selection, specifically testing whether selection is relaxed or intensified along a predefined set of ‘test’ (T) branches relative to a ‘reference’ (R) set. It builds on the branch-site random effects likelihood (BS-REL) framework to model site-specific selection pressures (ω, the dN/dS ratio) across different branches.

To quantify changes in selection strength, RELAX introduces a selection intensity parameter (*k*) that modifies the distribution of *ω* values in the test branches as a power function of the reference distribution: *ω*_T_ = *ω*_R_ᵏ

- If *k*<1, selection is relaxed: purifying selection becomes weaker and positive selection less intense.
- If *k*>1, selection is intensified: purifying selection becomes stronger and positive selection more intense.
- If *k*=1, selection strength is the same across the test and reference branches.

A likelihood ratio test (LRT) is used to compare the null model (*k*=1) against the alternative model (*k* estimated). A significant LRT result indicates a change in selection intensity. The method accounts for both shifts in the magnitude of *ω* values and changes in the proportion of sites under different selection regimes.

### Bacterial strains and culturing

Strains *Escherichia coli* HfrG6 (*ΔhisA*) and WFBG (*ΔtrpF*) (FBG Lab) [20] and *E. coli ΔhisG::kan* from the Keio collection [27] were used for complementation assays. For antibiotic production characterization, the strain *Nonomuraea* (*Actinomadura*) sp. ATCC-39365 was purchased from the ATCC repository. The strains *S. becharense* DSM 46887, *Microbispora triticiridacis* DSM 104649, *M. fusca* DSM 104648 and *Microbispora tritici* DSM 104650 were purchased from the DSMZ collections. The strain *Nonomuraea spiralis* DSM 43555 was a donation from NAICONS. All of the strains are stored in the FBG lab. Liophilized mycelium or fresh cultures were resuspended and cultured in N-Z amine medium (glucose 10 g l^−1^, soluble starch 20 g l^−1^, yeast extract 5 g l^−1^, N-Z amine type A 5 g l^−1^, CaCO_3_ 1 g l^−1^). Spore and mycelium stocks were prepared with glycerol 50% v/v.

### Genome sequencing and assembling

High molecular weight DNA was prepared following the standard protocols optimized for Oxford Nanopore Technologies long-read sequencing. Biomass for DNA extraction was obtained from *Nonomuraea* (*Actinomadura*) sp. strain ATCC-39365 cultures, which were eventually found to belong to the *Nonomuraea* genus (see below). Biomass was then flash-frozen with liquid nitrogen and ground in a mortar. This ground material was then resuspended in 5 ml of TE buffer (Tris 10 mM, EDTA 1 mM, pH 8); 250 µl of SDS 30%, 50 µl of RNAse (10 mg ml^−1^), 50 µl of proteinase K (20 mg ml^−1^) and 0.05 g of lysozyme from chicken egg white (Sigma) were added. The solution was incubated at 37 °C for 30 min, after which 1 ml of NaCl 5 M and 300 µl of CTAB/NaCl were added. The solution was incubated again at 65 °C for 20 min, and extraction was done with chloroform–isoamyl alcohol (24 : 1). DNA was then precipitated with isopropanol and resuspended in sterilized MilliQ water. DNA quality was checked by NanoDrop and gel electrophoresis. Raw reads were assembled *de novo* using Flye version 2.9 [28], resulting in a single contig corresponding to the complete genome of the strain. This sequence is available in the NCBI Assembly repository (BioProject: PRJNA1225233; BioSample: SAMN46880669).

### Functional complementation assays of HisA, HisG and TrpF activities

The *priA*, *adeK* and *trpF* genes from *Nonomuraea* (*Actinomadura*) sp. ATCC-39365, *M. fusca* DSM 104648*, S. becharense* DSM 46887 and *hisA* and *priA* from *O. murale* DSM 22056 were synthesized by Synbio Technologies (USA). The codons were optimized for heterologous expression in *E. coli*. These genes were synthesized into the pET28a vector (Novagen) and subcloned into the pASK (modified from pASK_IBA3plus) vector for complementation assays using NdeI and HindIII restriction sites under the *tet* promoter regulation expression. Protein expression was induced with anhydrotetracycline 20 ng ml^−1^. pASK vector is a high-copy-number plasmid containing F1 and ColE1 replication origins. It contains the *tetA* sequence, a strong promoter inducible with (anhydro)tetracyclin for heterologous expression. Previously, we have specially used pASK for complementation assays involving enzymes with very low promiscuous activities [29,30].

The *adeC* and *adeL* genes were amplified from *Nonomuraea* (*Actinomadura*) sp. ATCC-39365 genomic DNA using adapted primers (pASK_fwd: tgaaagcttgacctgtgaagtgaaaaatggcgc, pASK_rev: atggctgccgcgcggcac, AdaC_fwd: tggtgccgcgcggcagccatatgatctcgctcgccctgc, AdaC_rev: cttcacaggtcaagctttcatcaacccgcttcccccgtggc, AdeL_fwd: tggtgccgcgcggcagccatatggtctcgctcgcgctg, and AdeL_rev: cttcacaggtcaagctttcatcacggcgtgacgtccttc) following the Circular Polymerase Extension Cloning (CPEC) protocol [31]. The amplicons were mixed with the linearized vector pASK and Q5 HiFi DNA Polymerase. The assembly was done as described: initial denaturation, 98 °C/30 s; 15X (denaturation 98 °C/10 s, annealing 63°C/30 s, extension 72 °C/90 s); final extension, 120 s.

*In vivo* TrpF and HisA complementation assays were performed as described previously [20,21, 32]. Briefly, *E. coli* mutants HfrG6 (*ΔhisA*) and WFBG (*ΔtrpF*) were transformed with pASK constructions encoding *priA*, *adeK* and *trpF* genes. *E. coli* mutant *ΔhisG* was transformed with pASK constructions encoding the *adeC* and *adeL* genes. Transformed *E. coli* strains were plated in M9 medium (M9 salts 1X, glucose 0.2%, MgSO_4_ 1 mM, CaCl_2_ 0.1 mM and pH to 7.4). M9 medium supplemented with l-histidine (50 mg ml^−1^) and l-tryptophan (37 mg ml^−1^) was used to determine cell viability in the non-transformed mutants.

### Protein structure prediction and comparative analysis

Predicted protein structures were generated using AlphaFold version 2.0 [33] for the PriA, AdeK and SubTrpF2 sequences from representative members of the families *Streptosporangiaceae* and *Ornithinimicrobiaceae*. The resulting three-dimensional models were selected based on the confidence scores and used for subsequent structural comparisons. Structural alignments were performed using ChimeraX [34] with the crystal structures of *Mycobacterium tuberculosis* PriA complexed with PRFAR (PDB: 2Y88) and CdRP (PDB: 2Y85) as reference models. In addition, Jalview [35] was used to generate and visualize structure-informed multiple sequence alignments.

### Production, purification and LC-MS/MS analysis of nucleoside antibiotics

The production of related antibiotics was performed according to the method described by Tunac and Underhill [36], briefly, *Nonomuraea* (*Actinomadura*) sp. ATCC-39365 was maintained in liquid medium (1% starch, 1% lactose, 0.35% yeast extract and 0.65% malt extract) at 28 °C with shaking at 220 r.p.m. for 6 days (144 h). Fermentation broths were extracted with three volumes of n-butanol, and the crude extracts were subjected to HPLC and LC-MS analyses.

Metabolite profiling was performed using a Shimadzu Nexera X2 UHPLC system (PDA detector) coupled to a Shimadzu 9030 QTOF mass spectrometer with an ESI source and calibrant delivery system (CDS). Two microlitres of the sample were injected onto a Waters ACQUITY HSS C18 column (1.8 µm, 2.1×100 mm, 100 Å) at 30 °C and a flow rate of 0.5 ml min^−1^. The mobile phases were 0.1% formic acid in water (A) and acetonitrile (B), with the following gradient: 5% B (1 min), 5–30% B (9 min), 30–100% B (1 min), hold at 100% B (3 min) and re-equilibration at 5% B (3 min). The flow was directed to waste for the first 0.5 min and then to the MS for 13.5 min.

The PDA monitored 200–600 nm at 4.2 Hz with a 1.2 nm slit; flow cell temperature was 40 °C. MS calibration was performed with NaI (Shimadzu) and an ESI tuning mix (Sigma-Aldrich) introduced via the CDS for online post-run mass correction.

Samples were analysed in positive ion mode using data-dependent acquisition. Full MS scans (m/z 100–1700, 10 Hz, ID on) were followed by MS/MS of the top two ions (threshold, 1,500 counts; ID off), fragmented by CID at 20 eV. Dynamic exclusion was set to 1 s. Source parameters were interface voltage 4.0 kV, interface temp. 300 °C, nebulizer gas 3 l min^−1^ and drying gas 10 l min^−1^. CDS probe settings were 4.5 kV (positive mode) and nebulizer gas 10 l min^−1^.

## Results

### Identification of two PriA expansions in *Actinomycetota: Ornithinimicrobiaceae* and *Streptosporangiaceae*

We searched the occurrence patterns and evolutionary history of potential PriA expansions in *Actinomycetota*. In a first analysis, we explored a comprehensive *Actinomycetota* Genome DB (*n*=1,284 genomes) previously used to explore central enzyme families recruited in specialized metabolism at a large scale [4]. Using EvoMining, we focus on determining expansions of the central enzyme family PriA with seed sequences (Enzyme DB) from *Streptomyces*, *Corynebacterium* and *Nonomuraea,* adding as outgroup the HisA sequence of *A. ferrooxidans*. As a result, EvoMining revealed unique expansions in the *Streptosporangiaceae* and *Ornithinimicrobiaceae* lineages (Fig. S1, available in the online Supplementary Material).

Consequently, we enriched our analysis, adding all available genomes in August 2024 in the NCBI Assembly database of S*treptosporangiaceae* (*n*=212) families and *Ornithinimicrobiaceae* (*n*=45). Our final *Actinomycetota* Genome DB was augmented to 1,541 curated genomes, while the Enzyme DB was complemented with PriA sequences from *Streptosporangiaceae*. These analyses consistently identified PriA expansions, for the first time to our knowledge, distributed in the *Ornithinimicrobiaceae* and *Streptosporangiaceae* families (Fig. 2b). In the next sections, we will describe genomic and functional properties of the PriA expansions first in *Ornithinimicrobiaceae* and then in *Streptosporangiaceae*.

### The PriA expansion in *Ornithinimicrobiaceae* is related to l-tryptophan biosynthesis

In *Ornithinimicrobiaceae*, EvoMining identified the systematic co-occurrence of HisA and PriA homologues in 100% genomes of this family, corresponding to the *Ornithinimicrobium* and *Serinicoccus* genera. As we used PriA seed sequences for the expansions search, the homologues annotated as HisA were classified as ‘copy with unknown metabolic fate’ (Fig. S2). However, we did not find EvoMining hits for specialized metabolism. HisA and PriA homologues formed two well-supported, monophyletic clades (Fig. 2b). The HisA clade grouped closely with the rooted HisA homologue from *A. ferrooxidans* and other sequences annotated as HisA. In contrast, the PriA homologues from *Ornithinimicrobiaceae* clustered in a more divergent clade together with other sequences annotated as PriA. These phylogenetic relationships suggest that the *priA* gene originated and diverged from a lineage-specific duplication of *hisA* within *Ornithinimicrobiaceae*. To explore whether this expansion extends beyond *Ornithinimicrobiaceae* in phylogenetically related genomes, we searched for PriA expansions in the genomes of *Janibacter* spp., which, together with *Ornithinimicrobium* and *Serinicoccus*, conform to the *Intrasporangiaceae* family [37]. Only one copy was identified per genome in *Janibacter*, which was more similar to PriA (~50% identity by BlastP) than HisA (~40% identity by BlastP) sequences (Fig. S4). Thus, 100% of the *priA* duplications were unique to the *Ornithinimicrobiaceae* family. We searched for TrpF expansions using EvoMining with the same *Actinomycetota* genomic databases and TrpF sequences from *Streptomyces sviceus* and *Nonomuraea* sp. ATCCC-39365 as seeds. In all the *Ornithinimicrobiaceae* genomes, TrpF was absent.

#### PriA subfunctionalization to TrpF activity defines the SubTrpF2 enzyme family in *Ornithinimicrobiaceae*

For the co-occurring HisA/PriA context in *Ornithinimicrobiaceae*, we run complementation assays for the HisA and PriA enzymes from *O. murale* DSM 22056. We cloned *hisA* and *priA* genes into the pASK vector [30] and transformed the auxotrophic strains *E. coli* HfrG6 (*ΔhisA*) and WFBG (*ΔtrpF*). As expected, HisA displayed only ProFAR isomerase activity, thus rescuing l-histidine auxotrophy. In contrast, PriA could not rescue l-histidine auxotrophy but rescued l-tryptophan auxotrophy, suggesting PriA has subfunctionalized to PRA isomerase (TrpF) activity. Therefore, we named this PriA subfamily SubTrpF2 (Table 1, Fig. 2b).

#### Conserved active-site mutations underpin PriA subfunctionalization to TrpF activity in *Ornithinimicrobiaceae*

**Fig. 3.**
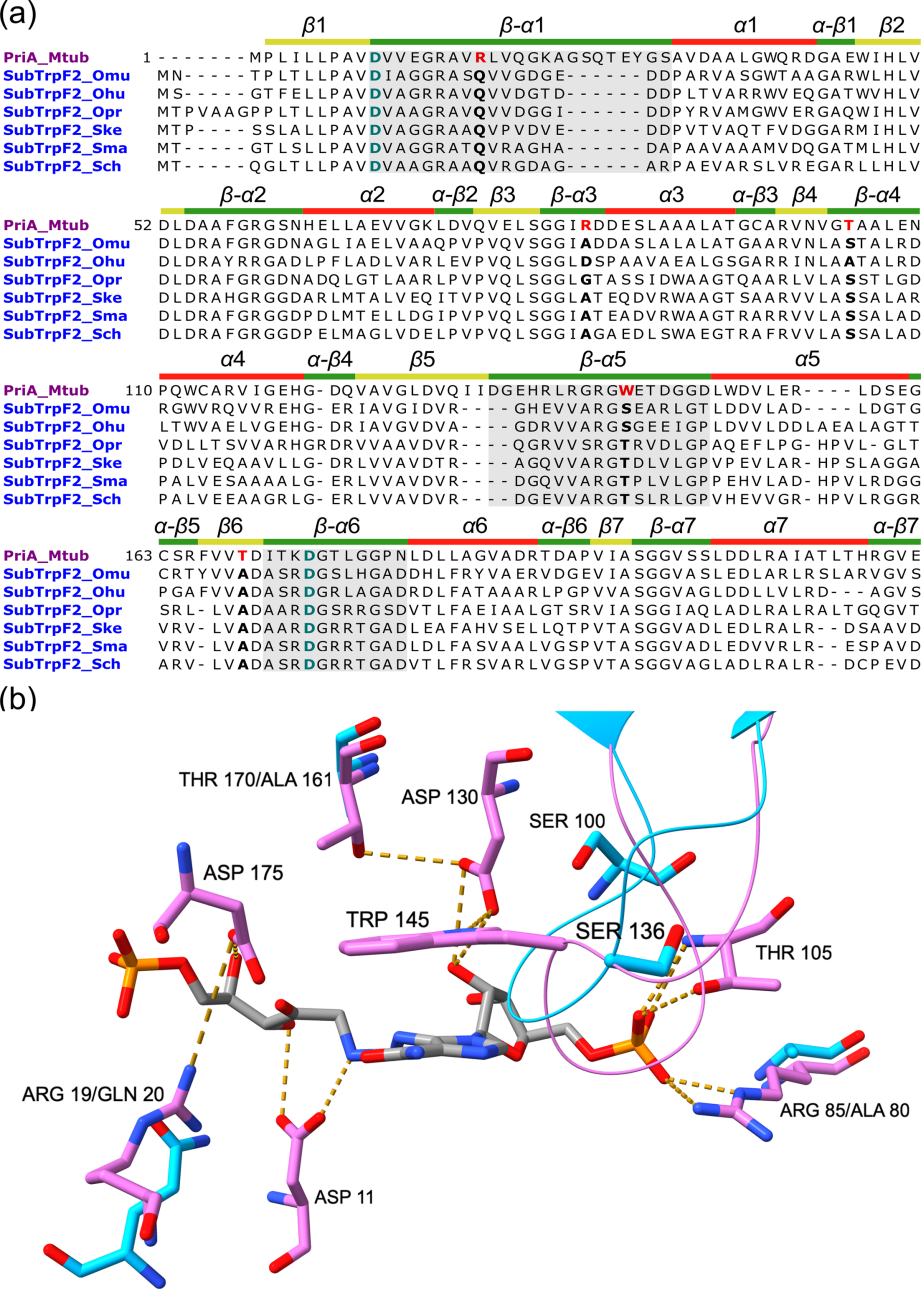
Sequence alignment of the SubTrpF2 family and structural analysis of the active site. (**a**) Multisequence alignment of PriA_Mtub (purple) and SubTrpF2 (blue) sequences of *Ornthinimicrobium and Serinicoccus* species. Catalytic residues Asp11 and Asp175 are coloured in blue–green. ProFAR binding residues are shown in red. Substitutions are shown in bold. The secondary structure is depicted above the sequence: loops in green, *α*-helices in red and *β*-sheets in yellow. Regions corresponding to loops 1, 5 and 6 are highlighted in grey. Position numbering was based on the PriA_Mtub sequence. (**b**) The structure of PriA_Mtub (violet) in a complex with PRFAR (PDB: 2Y88) superimposed with the AlphaFold predicted structure of SubTrpF2_Omu (cyan) is used to illustrate the position of the respective substrates. The conserved Asp11, Asp130 and Asp175 residues are only shown for PriA_Mtub. The ProFAR-binding residues are shown for both structures to visualize substitutions. For clarity, in SubTrpF2, Trp145 is substituted by Ser136, and Thr105 is substituted by Ser100. Loop 5 is shown as thin tubes.

To identify mutations that may be impairing the HisA activity in SubTrpF2, we predicted the three-dimensional structure of SubTrpF2 of *O. murale* (SubTrpF2_Omu) [33] and performed a structure-based alignment with *Mycobacterium tuberculosis* PriA complexed with the PRFAR substrate (PriA_Mtub; PDB: 2Y88) as reference (Fig. 3b). We complemented this analysis with a structure-informed multiple sequence alignment including PriA homologues from *Ornithinimicrobium humiphilum*, *Ornithinimicrobium pratense*, *Serinicoccus kebangsaanensis*, *Serinicoccus marinus* and *Serinicoccus chungangensis* (Fig. 3a).

Key residues Arg19, Arg85, Thr105, Trp145 and Thr170 were found to be changed with the same residues in all of the sequences. In SubTrpF2_Omu, the corresponding replacements were Arg19→Gln20, Arg85→Ala80, Thr105→Ser100, Trp145→Ser136 and Thr170→Ala161 (Fig. 3). Substitutions at these positions have been previously demonstrated to impair or abolish ProFAR Isomerization (HisA activity) by disrupting substrate binding and catalytic interactions in *Mycobacterium tuberculosis* and *Streptomyces coelicolor* PriA homologues [38,39]. In particular, mutations at Arg19 impede the substrate-specific recruitment of catalytic Asp175 to the active site. Trp145, together with Thr170, interacts with Asp130 through hydrogen bonds, stabilizing the substrate-specific knot-like conformation of loop 5. Such a structure positions TrpF145 inside the pocket to bind ProFAR through stacking interactions. Arg85 and Thr105 form the N-terminal phosphate-binding site that interacts with the second phosphate group of ProFAR, allowing its correct positioning in the substrate-binding pocket of PriA [38]. Overall, modifications at these residues in SubTrpF2 support the functional shift of SubTrpF2 towards TrpF activity and explain its inability to complement HisA function in the *E. coli ΔhisA* strain.

#### PriA expansions are recruited in a physiological genome context in *Ornithinimicrobiaceae*

In *Ornithinimicrobiaceae,* as expected, *hisA* copies are encoded within a *his* operon. However, unlike other *trp* operons in *Actinomycetota* [40], none of the *trp* genes were encoded in or near this region. Contrastingly, *subtrpF2* homologues are localized within a genomic context that, to our knowledge, has not been previously reported. Annotation of the adjacent genes in this region revealed functions essential for physiological processes, including glutamate/glutamine synthetase, bacilopeptidase F, tRNA acyl transferase, catalase and regulatory genes. The gene content and identity were highly conserved across the loci where PriA expansions were encoded in the genera *Ornithinimicrobium and Serinicoccus* (Fig. S7).

### The PriA expansion in *Streptosporangiaceae* is related to specialized metabolism

In *Streptosporangiaceae,* central PriA co-occurs with AdeK, its expansion in specialized metabolism, in 5.6% (*n*=11) of the explored genomes (*n*=212) (Fig. 2b, Table S1). This expansion is not uniformly distributed across *Streptosporangiaceae* genera. The family AdeK was distributed in the genomes as follows: 4.4% (*n*=3/67 genomes) of *Nonomuraea*, 14.28% (6/42 genomes) of *Microbispora* and 11.1% (2/18 genomes) of *Streptosporangium* (Table S1). EvoMining hits (green circles) are phylogenetically closer to a specialized recruitment from MIBiG (blue circle) (Fig. 2b**,** Fig. S2). This MIBiG hit corresponds to the phosphoribosyl isomerase AdeK encoded in the *ade* BGC previously characterized in *Nonomuraea* (*Actinomadura*) sp. ATCC-39365 [17,18] and shown to encode the biosynthetic pathways of adechlorin and 2′-amino dA. EvoMining hits, named here as the AdeK subfamily, were found across the genera *Nonomuraea*, *Microbispora* and *Streptosporangium*.

#### Phylogenetic and genomic evidence support the reclassification of *Actinomadura* sp. ATCC-39365 *as Nonomuraea* sp.

Strain ATCC 39365 was originally classified as *Actinomadura*; however, in the phylogenetic tree, its AdeK sequence forms a clade with homologous sequences from *Nonomuraea* genomes. In contrast, the *Actinomadura* genomes do not possess AdeK copies (Figs 2b and S2), and blastp searches on NCBI using AdeK as a query against *Actinomadura* genomes yielded only one hit per genome. We analysed the genome contexts of these homologues and realized that all of them were *priA* genes located within a *his* operon. These findings prompted us to sequence the ATCC-39365 genome (since only the *ade* BGC sequence was available) and compare it against the Genome Taxonomy Database (GTDB) [41], identifying its closest relative as *N. spiralis* (92.83% ANI). A *de novo* multilocus gene tree was constructed using all available *Streptosporangiaceae* genomes, placing the genome of strain ATCC-39365 firmly within the *Nonomuraea* clade, distinctly separated from the available *Actinomadura* genomes (Fig. S3). Based on this evidence, we will refer to the ATCC-39365 strain as *Nonomuraea* sp. ATCC-39365. Importantly, the genome sequence of *Nonomuraea* sp. ATCC-39365 allowed us to identify that *adeK* co-occurs with a *priA* homologue, which was not confirmed previously [17].

#### The TrpF family co-occurs with PriA and AdeK in *Streptosporangiaceae*

Since TrpF has one of the functional PriA activities, we wondered whether the *trpF* gene was present in the *Streptosporangiaceae* genomes. Although in the *Actinomycetota phylum*, *trpF* is regularly missing, it has been reported in genomes from the genera *Corynebacterium* and *Streptomyces* [20,21]. EvoMining analysis showed that the TrpF family was highly represented in *Streptosporangiaceae*, occurring in ~40% of genomes analysed (Table S1). While TrpF homologues were not found in *Microbispora*, EvoMining identified single-copy TrpF homologues, classified as central metabolism, broadly distributed in *Nonomuraea* and *Streptosporangium*. These lineages showed high PriA/TrpF co-occurrence frequencies of 97% and 83.3%, respectively, which is substantially higher than the low-frequency patterns previously reported in *Streptomyces* [21]. Interestingly, *trpF* is encoded in all of the *Nonomuraea* and *Streptosporangium* genomes containing *priA* and *adeK*, revealing a unique *priA/adeK/trpF* co-occurrence reported here for the first time. While no EvoMining hits were observed, this analysis showed an MIBiG hit, i.e. a *trpF* recruitment close to the BGC showdomycin, a nucleoside antibiotic produced by *Streptomyces showdoensis* [42] (Fig. S5).

#### PriA retains dual HisA/TrpF function, while AdeK specializes in HisA activity in *Streptosporangiaceae*

To elucidate the activities of the co-occurring homologues, we performed complementation assays. All sequences were synthesized for codon optimization and were subcloned into the pASK vector [30]. They were then tested in complementation assays in *E. coli* mutant strains with HisA or TrpF auxotrophy [HfrG6 (*ΔhisA*) and WFBG (*ΔtrpF*)]. To address the *priA/adeK/trpF* co-occurrence in *Streptosporangiaceae*, we cloned and tested the enzymes PriA, AdeK and TrpF from *Nonomuraea* sp. ATCC-39365, *S. becharense* DSM 46887 and *M. fusca* DSM 104648 (only PriA and AdeK; there is no *trpF* gene in this genome). In all cases, PriA showed consistent complementation of both HisA and TrpF activity. Interestingly, AdeK only rescued the growth of *ΔhisA* strains (Table 1). As expected, TrpF exhibited only PRA isomerase activity.

**Table 1.** Functional characterization of PriA and TrpF homologues in *Ornithinimicrobiaceae* and *Streptosporangiaceae*

Lineage	Organism	Enzyme	Abundance per genome by genus (%)	*In vivo activity*†	
				HisA	TrpF
***Ornitinimicrobiaceae***	*O. murale*	HisA	100	+	−
		SubTrpF2	100	−	+
***Streptosporangiaceae***	*Nonomuraea* sp. ATCC-39365	PriA	100	+	+
		AdeK	4.4	+	−
		TrpF	97.7	−	+
	*M. fusca* DSM 104648*	PriA	100	+	+
		AdeK	16.6	+	−
	*S. becharense* DSM 46887	PriA	100	+	+
		AdeK	11.1	+	−
		TrpF	83.3	−	+

* *Microbispora* genomes do not encode *trpF* genes. †*In vivo* activity tested heterologously in *E. coli *HfrG6 (*ΔhisA*) and WFBG (*ΔtrpF*) strains.

#### Conserved residue changes in the active site and relaxed purifying selection underlie the functional shift of AdeK towards HisA activity

**Fig. 4.**
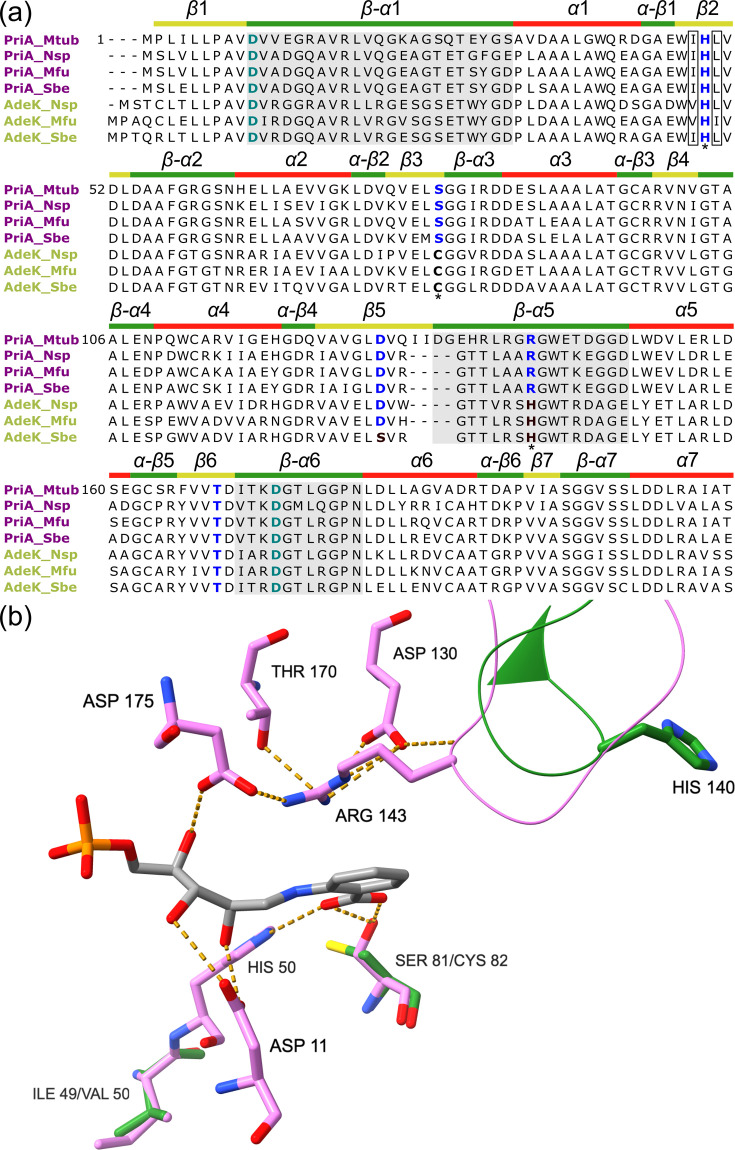
Sequence alignment of PriA and AdeK subfamilies and structural analysis of the active site. (**a**) Multisequence alignment of PriA (purple) and AdeK (blue) sequences of *Nonomuraea*, *Microbispora and Streptosporangium*. Catalytic residues Asp11 and Asp175 are coloured in blue-green. Residues relevant to the binding site for PRA are highlighted in blue. Substitutions are shown in bold. Loss-of-function substitutions are marked with an asterisk. The secondary structure is depicted above the sequence: loops in green, *α*-helices in red and *β*-sheets in yellow. Regions corresponding to loops 1, 5 and 6 are highlighted in grey. Position numbering was based on the PriA_Mtub sequence. (**b**) The structure of PriA_Mtub (violet) in a complex with CdRP (PDB: 2Y85) superimposed with the AlphaFold predicted structure of AdeK_Nsp (green) is used to illustrate the position of the respective substrates. The conserved Asp11, His50, Asp130, Thr170 and Asp175 residues are only shown for PriA_Mtub. The ProFAR binding residues are shown for both structures to visualize substitutions. For clarity, in AdeK, Arg143 is substituted by His140. Loop 5 is shown as thin tubes.

To identify mutations potentially responsible for the loss of TrpF activity in AdeK, we predicted the three-dimensional structure of AdeK from *Nonomuraea* sp. (AdeK_Nsp) using AlphaFold [33] and performed a structure-based alignment with PriA_Mtub complexed with the CdRP substrate (PDB: 2Y85) as reference (Fig. 4b). We complemented this analysis with a structure-informed multiple sequence alignment including PriA and AdeK homologues from *M. fusca* (PriA_Mfu, AdeK_Mfu) and *S. becharense* (PriA_Sbe, AdeK_Sbe) (Fig. 4a).

In PriA, residues Arg143, Ser81 and His50 are essential for PRA binding and catalysis [32,38]. In all AdeK homologues, Arg143 is replaced by histidine, potentially impairing the substrate-specific recruitment of Asp175 due to steric impediment, despite the retention of a positive charge. Furthermore, the Ser81→Cys82 substitution in AdeK alters a strictly conserved residue required for hydrogen bonding with the anthranilate moiety of PRA. Indeed, the analogous Ser81Thr mutation was previously shown to abolish PRA isomerization in PriA [32]. While His50 is conserved in AdeK, neighbouring residues Ile49 and Leu51 show substitutions to Val (AdeK_Nsp, Ade_Mfu) and Ile (Adek_Sbe), respectively, though their effects on catalytic activity remain uncertain. Altogether, these substitutions in AdeK homologues support a functional shift toward HisA activity and explain the enzyme’s inability to complement TrpF function in the *E. coli ΔtrpF* strain.

In addition, we hypothesized that subfunctionalization of AdeK was occurring in the context of the relaxation of purifying selection. Thus, we then analysed the PriA phylogenetic tree of *Streptosporangiaceae* using RELAX [26] (see the ‘Methods’ section). We defined the AdeK branch as the test branch and compared it with all PriA branches in the phylogenetic tree. RELAX found significant evidence of the relaxation of purifying selection in the AdeK branch (*P*=0.003, LR=9.04) (Figs S2 and S6).

#### PriA*–Streptosporangiaceae* expansions are recruited in the adechlorin BGC family

**Fig. 5.**
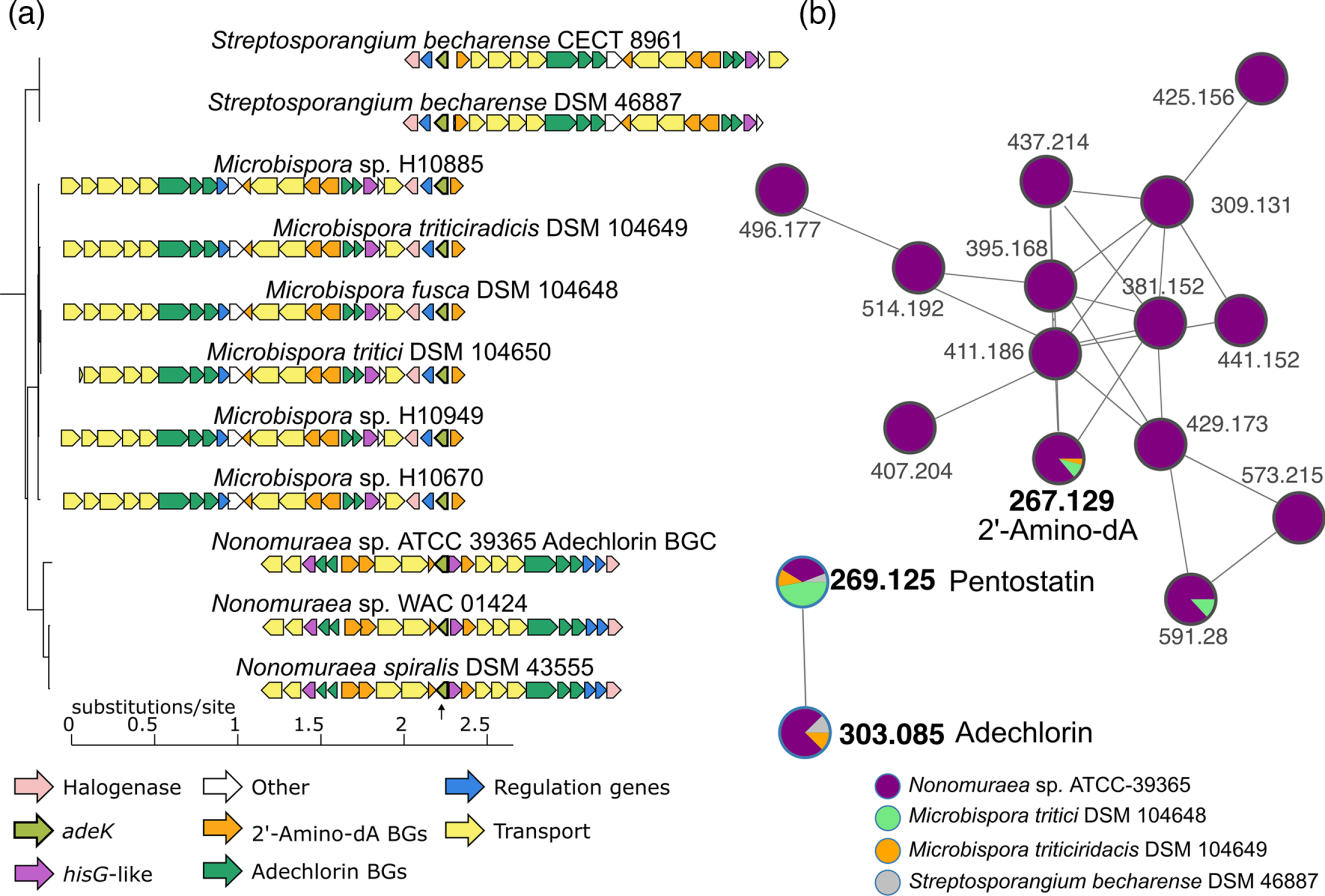
Recruitment of *adeK* into conserved Ade GCF and confirmation of nucleoside antibiotic production in *Streptosporangiaceae*. (**a**) CORASON analysis of *Streptosporangiaceae* genomes reveals *adeK* recruitment into conserved *ade* BGCs across *Nonomuraea*, *Microbispora* and *Streptosporangium. adeK* (light green) is highlighted with an arrow. (**b**) Molecular network from *Streptosporangiaceae* non-targeted MS data. MS analysis confirms the production of nucleoside antibiotics by *Nonomuraea* sp. ATCC-39365, *M. tritici* DSM 104650, *M. triticiridacis* DSM 104649 and *S. becharense* DSM 46887.

The conservation of genomic neighbourhoods encoding *priA* homologues and *adeK* was analysed using CORASON [25]. Genomes revealed that 100% of *priA* genes from central metabolism were recruited in the l-histidine and l-tryptophan biosynthetic genome contexts, consistent with their conserved role in the biosynthesis of these amino acids. As mentioned, *adeK* is recruited to the *ade* BGC (Fig. 5a). This was anticipated for *Nonomuraea* sp. ATCC-39365 [17,18]. However, CORASON results indicated that *adeK* homologues are recruited in genome contexts that maintain high gene identity (>64% identity) with reference *ade* BGC sequences (Fig. 5a). These analyses revealed a group of taxonomically related BGCs, named here as the Ade gene cluster family (GCF), distributed across *Streptosporagiaceae*, similar to the *adeK* gene family*,* comprising 11 BGCs: 3 in *Nonomuraea*, 6 in *Microbispora* and 2 in *Streptosporangium*. The gene composition of the *ade* BGC is conserved, as *S. becharense* DSM 46887 and *M. fusca* DSM 104648 conserve 19 and 20 homologous genes, respectively, from the *Nonomuraea* sp. ATCC-39365 *ade* BGC.

We next evaluated whether the micro-organisms belonging to the Ade GCF could produce the predicted compounds. To answer this question, we grew *N. spiralis* DSM 43555, *M. tritici* DSM 104650, *M. fusca* DSM 104648, *M. triticiridacis* DSM 104649 and *S. becharense* DSM 46887 on standard antibiotic production fermentations [36]. We performed parallel fermentation experiments using *Nonomuraea* sp. ATCC-39365 as a positive control for antibiotic production. We extracted the supernatant and performed an untargeted metabolomic analysis. LC-MS data were used to build GNPS networks to visualize and annotate the related molecules based on their spectral similarity [43]. Molecular networking analysis revealed that pentostatin and adechlorin spectra clustered together within a distinct two-node molecular network, while the spectrum corresponding to 2′-amino dA was found within a separate, densely populated molecular network. As previously reported [17], all three compounds (i.e. 2′-amino dA, adechlorin and pentostatin) were detected in extracts of *Nonomuraea* sp. ATCC-39365. In addition, we confirmed that *M. tritici* DSM 104650 and *M. triticiridacis* DSM 104649 also produced 2′-amino dA and pentostatin, although at lower titres. Pentostatin production was also observed in *S. becharense* DSM 46887. Interestingly, adechlorin was detected in all of these strains except for *M. tritici* DSM 104650. In contrast, neither *N. spiralis* DSM 43555 nor *M. fusca* DSM 104648 produced detectable levels of any of the three compounds under the conditions tested (Fig. 5b). Even when these BGCs are highly similar, specific physiological and environmental factors related to each strain might therefore play a role in the production of adechlorin and 2′-amino dA. Further characterization and standardization efforts will be needed to find the specific expression conditions for these strains.

#### Biosynthetic genes of adechlorin are shared with the pentostatin, coformycin and pyrazomycin BGCs

**Fig. 6.**
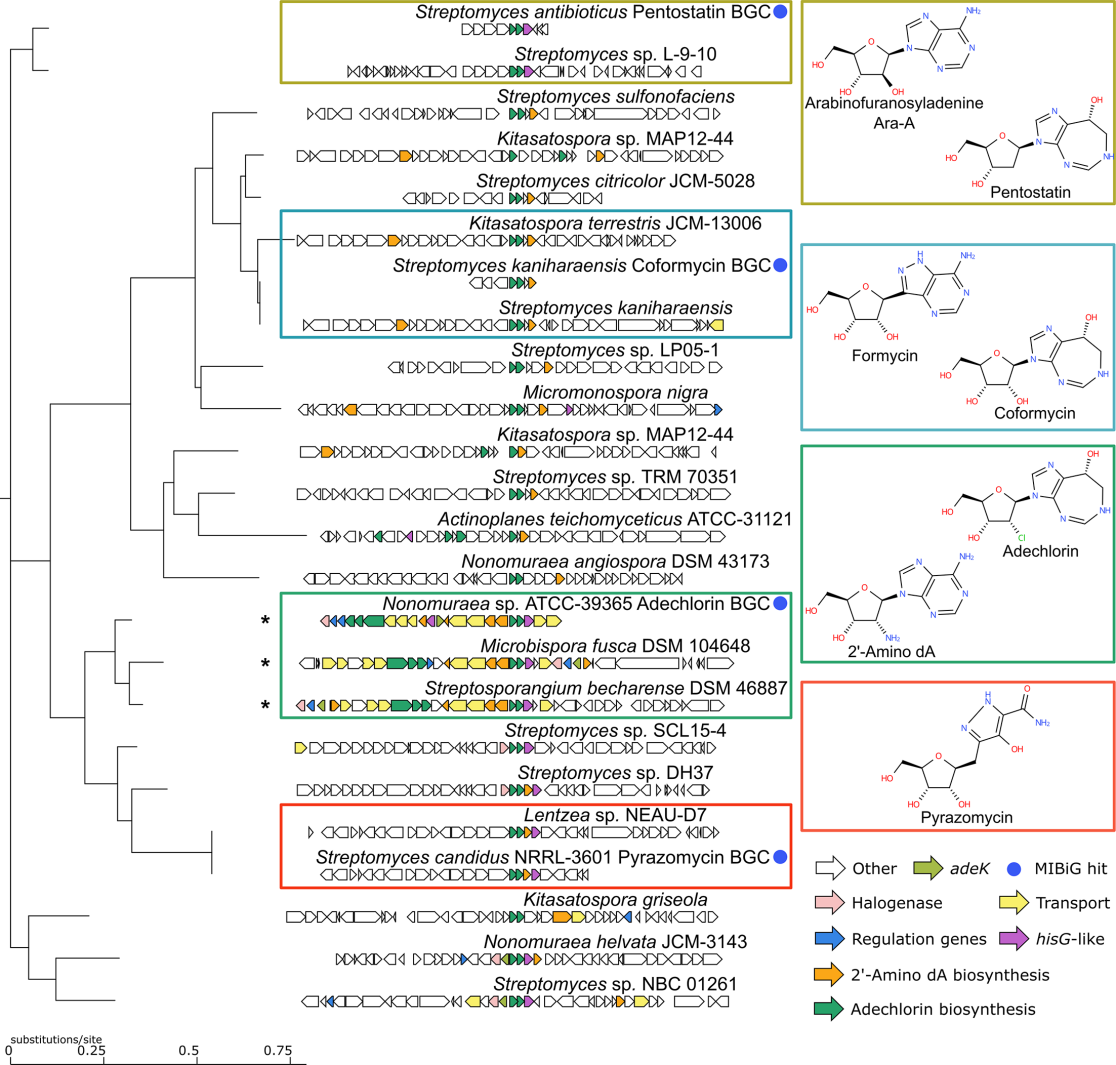
CORASON phylogeny of nucleoside antibiotic BGCs in *Actinomycetota*. CORASON phylogenetic reconstruction with *adeA* as the query gene and the *Nonomuraea* sp. ATCC-39365 *ade* BGC, as the cluster reference, depicts the clades of pentostatin/Ara-A, coformycin/formycin, adechlorin/2′-amino dA and pyrazomycin GCFs, as well as unknown related BGCs. The nucleoside antibiotic structures illustrate the correspondence between genomic and molecular variation. Reference MIBiG BGCs are indicated with blue dots. The micro-organisms tested for nucleoside antibiotic production are marked with asterisks.

To investigate in a broader context the evolutionary recruitment of *adeK* into its BGC, we performed a CORASON-based phylogenomic analysis of 25 representative *Actinomycetota* genomes. Using *adeA*, a core biosynthetic gene that encodes SAICAR synthetase, as a query, and the *ade* BGC as a reference, we reconstructed the phylogenetic tree of nucleoside antibiotic BGCs. These analyses reveal four major clades corresponding to known biosynthetic pathways: pentostatin, coformycin, pyrazomycin and adechlorin (Table S3, Fig. 6). Each clade included both reference and previously uncharacterized BGCs, expanding the known taxonomic distribution of these pathways. For example, coformycin BGCs were found in *Kitasatospora terrestris* and pyrazomycin BGCs in *Lentzea* species. Regarding the uncharacterized genome contexts, we identified *adeV* halogenase homologues in multiple *Streptomyces* BGCs, suggesting novel halogenated nucleoside antibiotic pathways.

The *adeC* and *adeL* genes are located within the *ade* BGC of *Nonomuraea* sp. ATCC-39365. They are homologues of *hisG*, which encodes an ATP phosphoribosyl transferase in histidine biosynthesis. The AdeC and AdeL amino acid sequences share 27% and 28% identity, respectively, with *hisG*. Functional complementation assays in *E. coli ΔhisG* strains demonstrated that neither AdeC nor AdeL could rescue l-histidine auxotrophy, indicating that these enzymes have a highly restricted role in adechlorin biosynthesis. However, only *adeC* homologues are conserved in the rest of the *ade* BGCs in addition to the pentostatin (i.e. *penA*) and pyrazomycin (i.e. *pyrS*) BGCs (Table S3).

While all *adeK* genes described above are only recruited in the Ade GCF, it is worth noticing the co-recruitment of *adeK* and *adeC* homologues in *Nonomuraea helvata* JCM 3143 and *Streptomyces* sp. NBC 01261 in non-*ade* BGC or *his* operon contexts. These findings, which escaped our initial genome mining analysis, suggest that co-recruitment of *adeK* and *adeC* represents a broader evolutionary strategy not restricted to the Ade GCF.

## Discussion  

This study expands our understanding of the evolutionary dynamics that shape enzyme multifunctionality and recruitment in *Actinomycetota*, a phylum renowned for its biosynthetic versatility and rich natural product repertoires. Focusing on the PriA family, we describe two distinct evolutionary trajectories arising from rare gene expansion events: (i) the co-occurrence of PriA and HisA in the *Ornithimicrobiaceae* family, with subfunctionalization to TrpF activity in PriA, and (ii) the emergence of a PriA homologue, i.e. AdeK, with subfunctionalization to HisA activity and potential substrate specialization within the *ade* BGC in members of *Streptosporangiaceae*.

PriA is a promiscuous enzyme encoded by a highly conserved single-copy gene in which subfunctionalization cases have occurred under specific dynamics. This enzymatic system aligns with the concept of ‘frustrated enzymes’, where trade-offs between multiple catalytic activities limit optimization [44]. While gene duplication can alleviate such constraints by enabling specialization [45], the low frequency of *priA* duplications observed in only 6.2% of the analysed genomes suggests that such events are evolutionarily rare and likely occur only under specific selective pressures.

On the one hand, the fully conserved co-occurrence of HisA and a divergent PriA homologue (i.e. SubTrpF2) in the deep-rooted *Ornithinimicrobiaceae* likely originated in the common ancestor of *Ornithinimicrobium* and *Serinicoccus* and was vertically inherited. Functional assays confirmed that SubTrpF2 retains only TrpF activity, while *hisA* encodes canonical ProFAR isomerase function. We previously described SubTrpF homologues encoded within the *his/trp* genome vicinity in genomes exhibiting gene-loss signs [19]. In contrast, SubTrpF2 homologues originated through an independent evolutionary trajectory, as their encoding genes are not found in a *his/trp* biosynthetic context and instead are located adjacent to genes linked to physiological metabolism and stress response. The conservation of this specialized TrpF function in SubTrpF2 suggests an adaptation, linked to broader physiological processes encoded at this locus, but further biochemical and functional characterization will be essential to elucidate the specific role of SubTrpF2 within this distinct genomic and metabolic framework.

On the other hand, the expansion and recruitment of PriA homologue, AdeK, in the genera *Nonomuraea*, *Microbispora* and *Streptosporangium* suggest lineage-specific evolution. First described within the *ade* BGC of *Nonomuraea* sp. ATCC-39365, which encodes the adechlorine and 2′-amino dA biosynthetic pathways [17]. Two ‘nucleoside antibiotics’ with utility in anticancer and antiviral treatments, respectively [46,47]. Our phylogenomic analysis revealed that *adeK* homologues are part of the BGCs sharing over 64% of core genes, suggesting a common evolutionary origin as a locus, but are only present in a few strains. Metabolomics analysis further confirmed the production of adechlorine, 2′-amino dA and pentostatin from some of the *ade* BGC-encoding strains (Fig. 5b). Functional assays and sequence analysis showed that AdeK has undergone subfunctionalization from an ancestral PriA enzyme, retaining only ProFAR isomerase activity due to loss-of-function mutations in conserved PRA-binding residues (Fig. 4).

Several studies have shown that duplicated genes are typically under purifying selection, although selection may be substantially relaxed relative to non-duplicated orthologs. This allows duplicated genes to diverge and creates the potential to give rise to new activities [48,49]. In line with this, we found significant evidence of the relaxation of purifying selection in the AdeK branch relative to the PriA branch. The co-occurrence of PriA, AdeK and TrpF may have increased the exploration of the sequence space in the interplay between optimization of metabolic flux and AdeK neofunctionalization, keeping the core mechanism of HisA activity but potentially exploring new affinities. Notably, the initial reactions of adechlorin biosynthesis are analogous to those of l-histidine biosynthesis [17], although the starting substrate 2′-Cl-2′-deoxyadenosine monophosphate [18] differs from ATP as in the canonical l-histidine pathway. Together with the evidence of AdeK’s HisA activity, we hypothesize that subsequent intermediates in the adechorine biosynthesis are chlorinated, including the one channelled to AdeK, possibly a ProFAR-like chlorinated analogue (Fig. 1). Although relaxed selection does not necessarily imply specialization, our results reveal patterns of relaxed selection, subfunctionalization and conservation of *adeK* within a shared genomic context. Nonetheless, characterization and confirmation of the substrate structure and catalysis mechanisms will be essential to elucidate its catalytic role in nucleoside antibiotic biosynthesis.

In addition, our findings reveal the *hisG* homologues (*adeC* and *adeL*) into the *ade* BGC, as well as into the pentostatin (i.e. *penA*) and pyrazomycin (i.e. *pyrS*) BGCs (Table S3). HisG condenses phosphoribosyl pyrophosphate (PRPP) and ATP in the l-histidine biosynthesis. Functional complementation assays in *E. coli ΔhisG* strains showed that neither AdeC nor AdeL restored HisG activity, indicating that both enzymes have undergone substantial functional specialization. This specialization may involve the loss of ATP-binding capacity to prevent interference with l-histidine biosynthesis, while enabling the alternative condensation of PRPP and 2′-Cl-2′-deoxyadenosine monophosphate substrates. The recruitment of *adeC* and *adeL* in the *ade* BGC likely provided a fitness advantage by supplying precursor molecules within co-evolving biosynthetic contexts, such as l-histidine, l-tryptophan, adechlorin and 2′-amino-2′-dA. Co-recruitment of en bloc *hisG* and *priA* homologues in the Ade GCF and uncharacterized BGCs in *N. helvata* JCM 3143 and *Streptomyces* sp. NBC 01261 suggests a broader evolutionary mechanism for reshaping and integrating central metabolic functions into specialized pathways.

Similar trends have been observed in the scytonemin pathway, a cyanobacterial sunscreen compound, where glutamate dehydrogenase and acetolactate synthase homologues have evolved new substrate specificities. The reactions catalysed by their central metabolic ancestors are limited to l-glutamate and pyruvate, respectively. In contrast, the scytonemin biosynthetic genes have broadened their substrate specificities: the *gdh* homologue *scyB* accepts l-tryptophan, and the *ilvB* homologue *scyA* accepts indole-3-pyruvate (I-3Py; the product of the reaction catalysed by ScyB) and p-hydroxy-phenyl-pyruvic acid (p-HPP). Mutations in expansions involving l-tryptophan and p-HHP precursor supply are apparent in the scytonemin BGC, including *aroB*, *aroG*, *tryA*, *trpE/G*, *trpD*, *trpC*, *trpA* and *trpB*, which likely play an essential role in the metabolic flux to modulate the availability of these key precursors [4,50, 51].

The family *Ornithinimicrobiaceae* comprises the *Ornithinimicrobium* and *Serinicoccus* genera. They exhibit ecological versatility, having been isolated from diverse environments such as soils, marine macroalgae, cave sediments and biofilm-colonized surfaces [52,55]. Few specialized metabolites have been characterized in this family. For instance, *Serinicoccus* sp. and *Serinicoccus profundi* have been shown to produce seriniquinone and 3-((6-methylpyrazin-2-yl)methyl)-1H-indole (cytotoxic and antibiotic activities, respectively) [56,57], and extracts from *Ornithinimicrobium kibberense* have demonstrated cytotoxic activity against mammalian cancer cell lines [58]. Although the abundance and diversity of BGC in this family have not been formally characterized, to our knowledge, the growing number of available genomes represents an underexplored resource for investigating their metabolic potential through integrative genome mining and metabolomic approaches.

On the other hand, the family *Streptosporangiaceae* comprises diverse genera[59] such as *Streptosporangium[60]*, *Microbispora*[59,60], *Nonomuraea, Planotetraspora* and *Lentzea*. With a remarkable ecological versatility, they are primarily isolated from terrestrial oligotrophic or extreme environments as well as from plant-associated environments. Members of this family are often regarded as ‘rare actinomycetes’ [61,65]. The *Streptosporangiaceae* family exhibits exceptional biosynthetic potential, with large genomes (≈12 Mb) encoding 25–35 BGCs per genome [66,67]. Several relevant antibiotics originate from this family, including the glycopeptide A40926, a dalbavancin precursor (*Nonomuraea sp*. ATCC-39727) [68]; microbisporicin (*Microbispora sp*. ATCC PTA-5024) [69]; and funisamine (*Streptosporangium sp*. KDCAGE35) [70]. Moreover, genome mining analysis has revealed abundant PKS, NRPS, terpene and RiPP BGCs [71], underscoring the untapped potential of this lineage for natural product discovery through integrative genomic and metabolomic approaches.

The contrasting genomic and biosynthetic features of *Ornithinimicrobiaceae* and *Streptosporangiaceae* provide an informative framework for interpreting the evolutionary scenarios uncovered for *priA* and *adeK*. In *Ornithinimicrobiaceae*, a relatively rare and taxonomically narrow actinobacterial family, secondary metabolite potential has been explored only in a few strains. The observation that PriA co-occurs with HisA, with concomitant subspecialization to TrpF activity and recruitment to a physiological genome context, seems to reflect a case of reliance on expansion of central metabolic capabilities that provides metabolic robustness and facilitates the evolution of new physiological roles and adaptation.

By contrast, *Streptosporangiaceae* have shown the potential to produce diverse natural products. Within this context, the recruitment of *priA* homologues into specialized metabolism, as exemplified by *adeK* in the *ade* BGC, illustrates a distinct evolutionary trajectory. Here, central metabolic enzymes are co-opted en bloc into complex BGCs, where they evolve restricted substrate specificities tailored to specialized metabolites. The identification of *adeK* across multiple *Streptosporangiaceae* genera underscores the case where gene family expansion provides metabolic robustness that may facilitate the evolution of specialized compounds as a key adaptive feature for highly competitive environments, which is consistent with *Streptosporangiaceae*’*s* large biosynthetic capacity and ecological success.

Our findings highlight two contrasting scenarios of PriA functional shift in central and specialized metabolism driven by gene family expansion in rare and underexplored actinobacterial taxa. More broadly, this work underscores the power of evolutionary genome mining to uncover novel biosynthetic potential, identify previously unrecognized natural product producers and elucidate the evolutionary logic underlying enzyme innovation in nucleoside antibiotic biosynthesis. These insights lay the foundation for future work aimed at biochemically characterizing additional PriA homologues and investigating their structural basis for substrate specificity, as well as the discovery of novel metabolites with potential new bioactivities.

## Supplementary material

10.1099/mgen.0.001634 Uncited Supplementary Material 1.

## Abbreviations

BGCs: biosynthetic gene clusters
CdRP: 1-(O-carboxyphenylamino)-1′-deoxyribulose-5′-phosphate
CDS: calibrant delivery system
CVD: Cluster Variation Database
DB: database
GCF: gene cluster family
LRT: likelihood ratio test
NCBI: National Center for Biotechnology Information
NP DB: Natural Product database
PRA: N-(5′-phosphoribosyl)-anthranilate
PRFAR: N-[(5-phosphoribulosyl)formimino]-5-aminoimidazole-4-carboxamide ribonucleotide
PriA: phosphoribosyl isomerase A
ProFAR: N-[(5-phosphoribosyl)formimino]-5-aminoimidazole-4-carboxamide ribonucleotide
PRPP: phosphoribosyl pyrophosphate
