# Supplementary material for: Functional shifts of the dual-substrate phosphoribosyl isomerase PriA from primary to specialized metabolism in rare Actinomycetota
Source: Microb Genom. 2026 Apr 7;12(4):001634. doi: 10.1099/mgen.0.001634 (PMC13055916; doi:10.1099/mgen.0.001634)
Supplement: Uncited Supplementary Material 1. [file mgen-12-01634-s001.pdf]

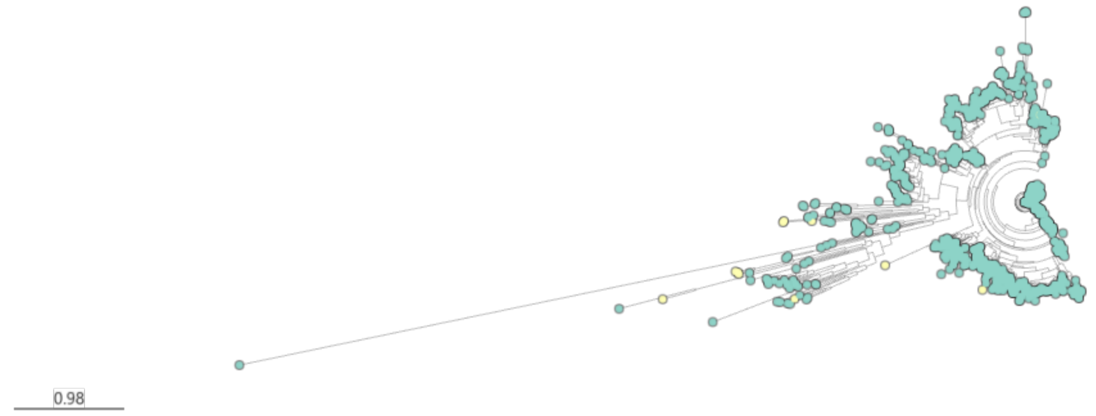

Figure S1 Preliminary PriA EvoMining Analysis in *Actinomycetota* genomes. Evomining PriA phylogeny of the *Actinomycetota* phylum shows most of the sequences restricted to singletons (blue), while a few expansions (yellow) can be seen across unrelated clades. Full tree and metada are available in the link https://microreact.org/project/7g2IGfkv9

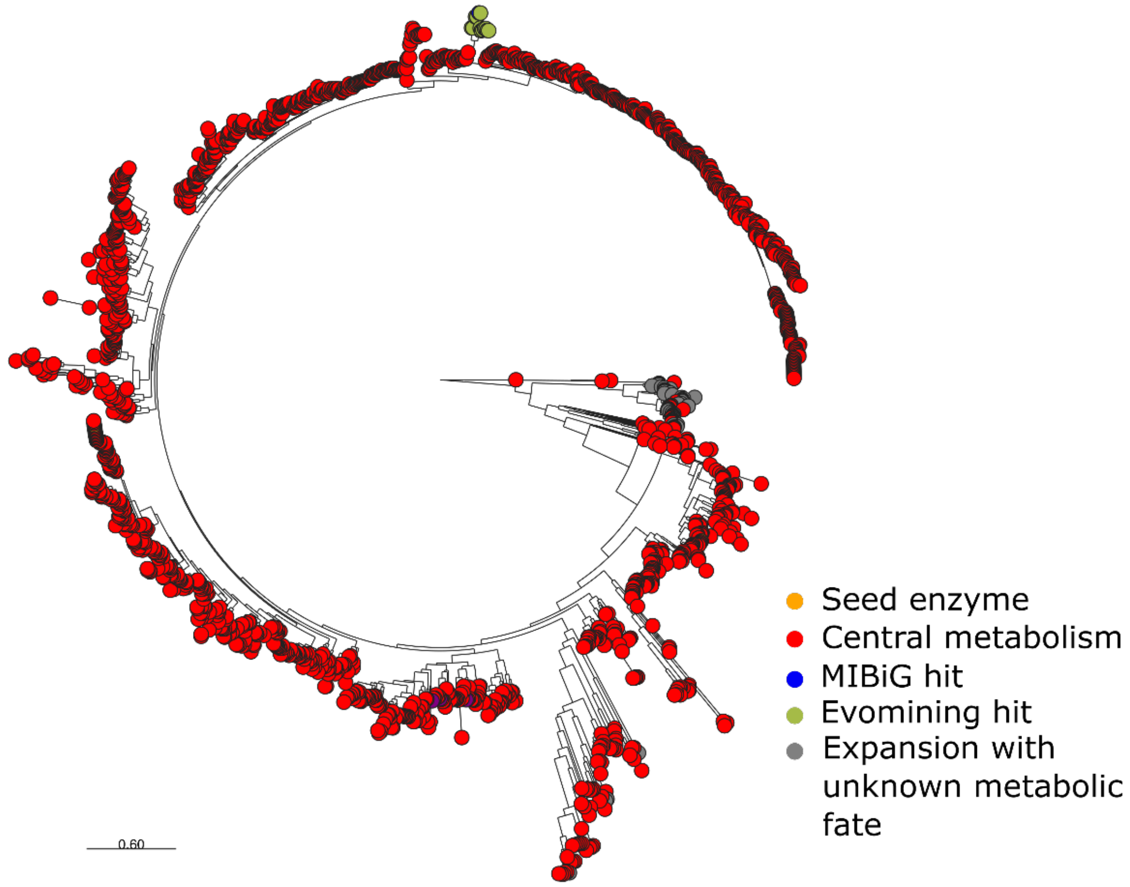

Figure S2. EvoMining analysis of PriA homologs in a expanded *Actinomycetota* genome database. EvoMining Phylogenetic reconstruction of PriA homologs across the Actinomycetota phylum, reveals their metabolic fates. Most homologs are confined to central

metabolism. However, two distinct expansion clades are evident. The clade marked with gray dots corresponds to HisA-like copies found in *Ornithinimicrobiaceae*, while the clade highlighted with green dots represents AdeK homologs (Evomining hits) associated with specialized metabolism. Full tree and metadata are available in the link:
https://microreact.org/project/67oryiUmNnujm3GLTudMuA-priaevomining110725paper

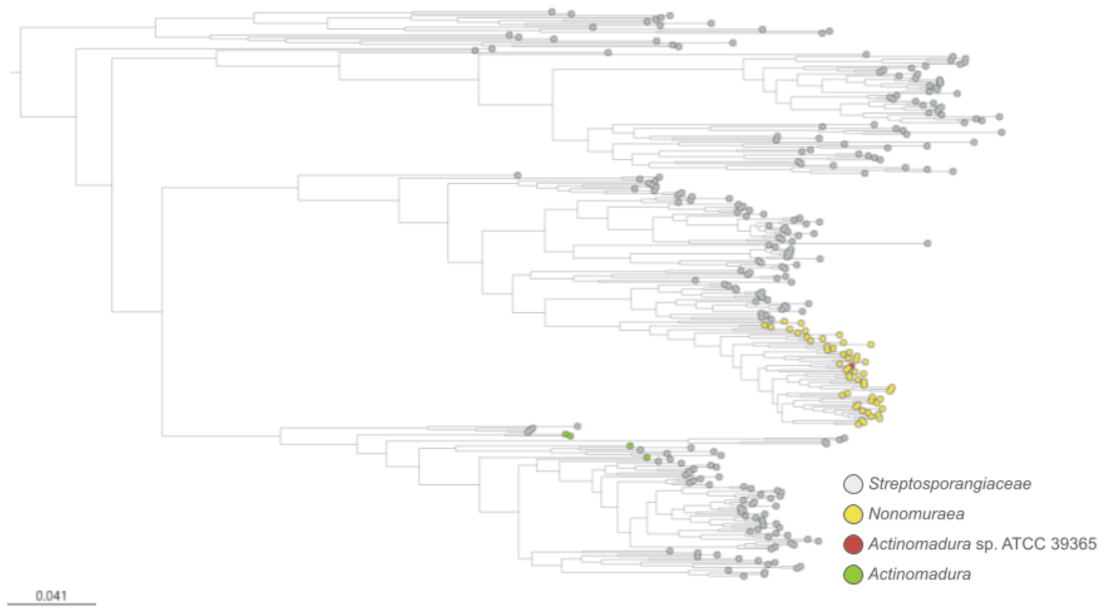

Figure S3. Phylogenetic placement of *Actinomadura sp.* ATCC-39365 within the *Nonomuraea* clade. The multilocus phylogenetic tree based on concatenated housekeeping genes reveals that *Actinomadura* sp. ATCC 39365 clusters within the *Nonomuraea* clade, rather than the *Actinomadura* clade. This placement supports its reclassification and indicates a closer phylogenetic relationship with *Nonomuraea*. Full tree and metadata available in the link:
https://microreact.org/project/eehGs4FhkcivVBhkwVDKhu-actinomadura-gtdbtk

Table S1. Distribution and co-occurrance frequencies of *priA* expansions and t*rpF* homologs in *Streptosporangiacea* and *Ornithinimicrobiaceae.*

**Lineage**	**Genera total genomes**	***Genomes with priA***	***Genomes with priA and trpF***	***Genomes with priA and no trpF***	***Genomes with trpF***	***Genomes with trpF* but no *adeK***	***Genomes with trpF* and *adeK***	***Genomes with adeK* but no *trpF***	***Genomes with adeK***
Streptosporangiaceae<br><br>(220/347= 40.89%)<br><br>N=212 Genomes	*Nonomuraea (N=67)*	67	65<br>97%	2	65	62	3	0	3<br>4.4%
	Microbispora (N=42)	42	0	42	0	0	0	6	6<br>14.28%
	Streptosporangium (N=18)	18	15<br>83.3%	3	15	13	2	0	2<br>11.1%
	Other *Streptosporangiaceae (N=85)*	84	25	59	25	25	0 ***	0	0
Total	*212*	211<br>99.5%	105<br>49.5%	106<br>50%	105<br>49.5%	100<br>47.1%	5<br>2.3%	6<br>2.83%	11<br>5.18%
Ornithinimicrobiaceae (0/45)	45	45<br>100%	Not found	Not found	Not found	Not found	Not found	Not found	Not found

*** *Actinomadura* sp. ATCC_39365 has *trpF* and *adeK*, but was reclassified as *Nonomuraea*

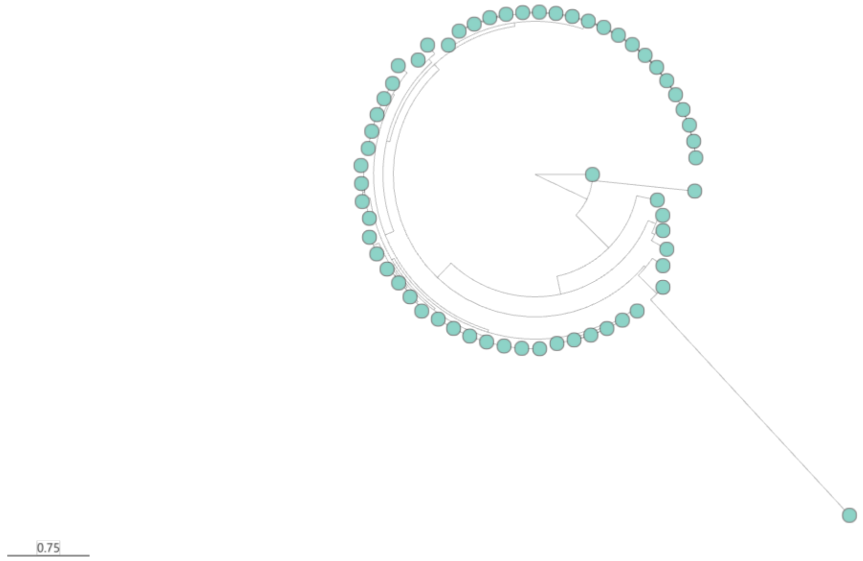

Figure S4. *Janibacter* genomes do not contain PriA expansions. Evomining phylogenetic analysis of *Janibacter* PriA homologs reveals only single-copy orthologs (blue). This contrasts with patterns observed in *Ornithinimicrobiacea* (see results). Full tree and metadata are available in the link:
https://microreact.org/project/ejQKbvWmVg5QbDYCyG2Fxx-priajanibacter

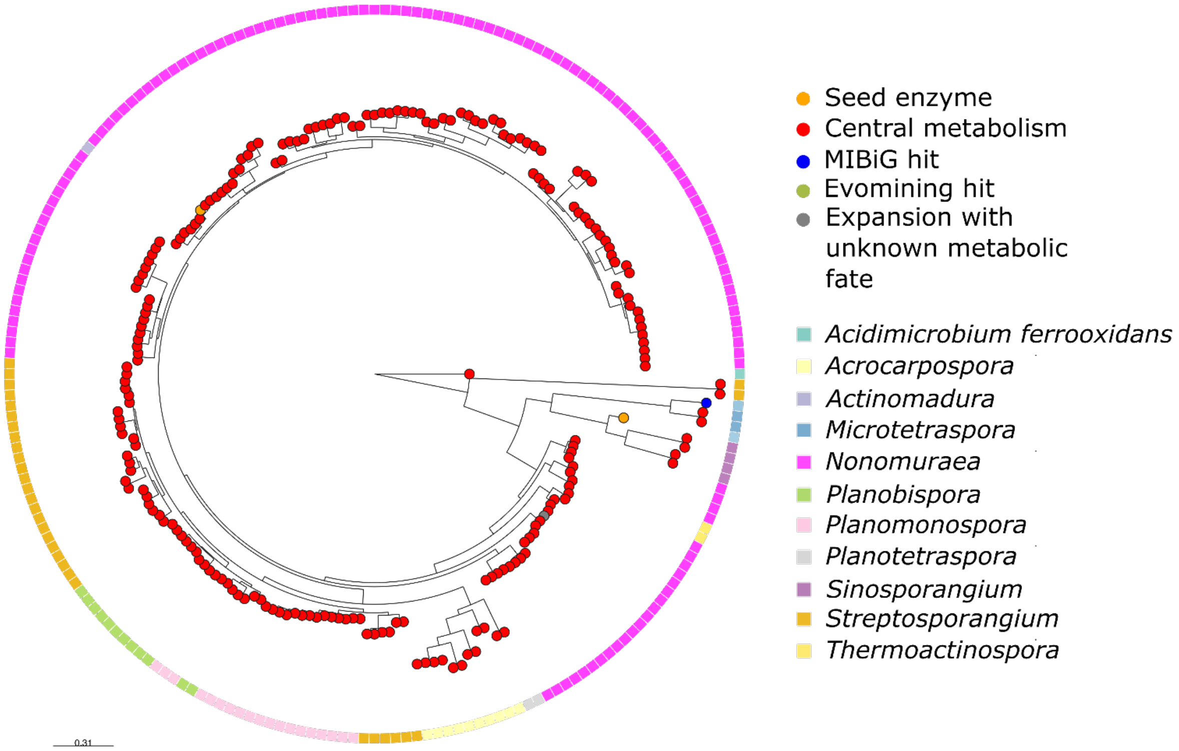

Figure S5. TrpF homolog are maily distributed in *Streptosporangiacea* genomes. Evomining phylogenetic reconstruction of *Actinomycetota* TrpF homologs shows that almost all of the TrpF copies are mainly in *Streptosporangiacea* genomes as single copy and labeled as central metabolism. A single MIBiG-associated homolog (blue) corresponds to a *trpF* gene encoded within the showdomycin biosynthetic gene cluster, indicating recruitment into specialized metabolism. Additionally, one homolog is classified with an "unknown metabolic

fate" (grey), lacking clear association with either central or specialized metabolic pathways. The full tree and metadata are available in the link: https://microreact.org/project/hz4g6FAqxmvxFUE3eVBSKt-trpfevominingrerooted

Table S2. RELAX values for the *adeK* test.

k	p	LR
0.66[a]	0.003[b]	9.04

[a] *k* < 1 means the purifying selection is relaxed.

[b] *k* estimation is statistically significant at the p < 0.01 level.

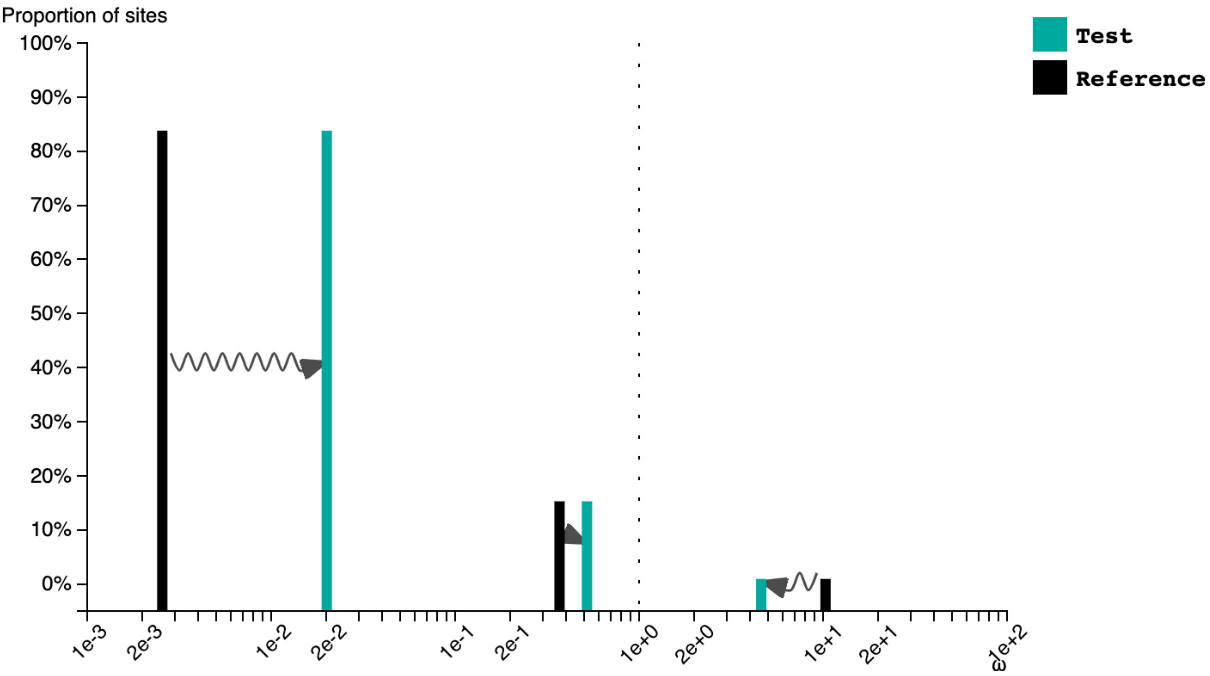

Figure S6. The *adeK* branch shows a pattern of significant relaxation of natural selection compared to the *priA* branch under the partitioned descriptive model. Three ω parameters and their relative proportion of sites are plotted for *adeK* (test, green) and *priA* (reference, black) branches. Only ω categories representing non-zero proportions of sites are shown. The gray vertical dashed line at ω = 1 represents neutral evolution.

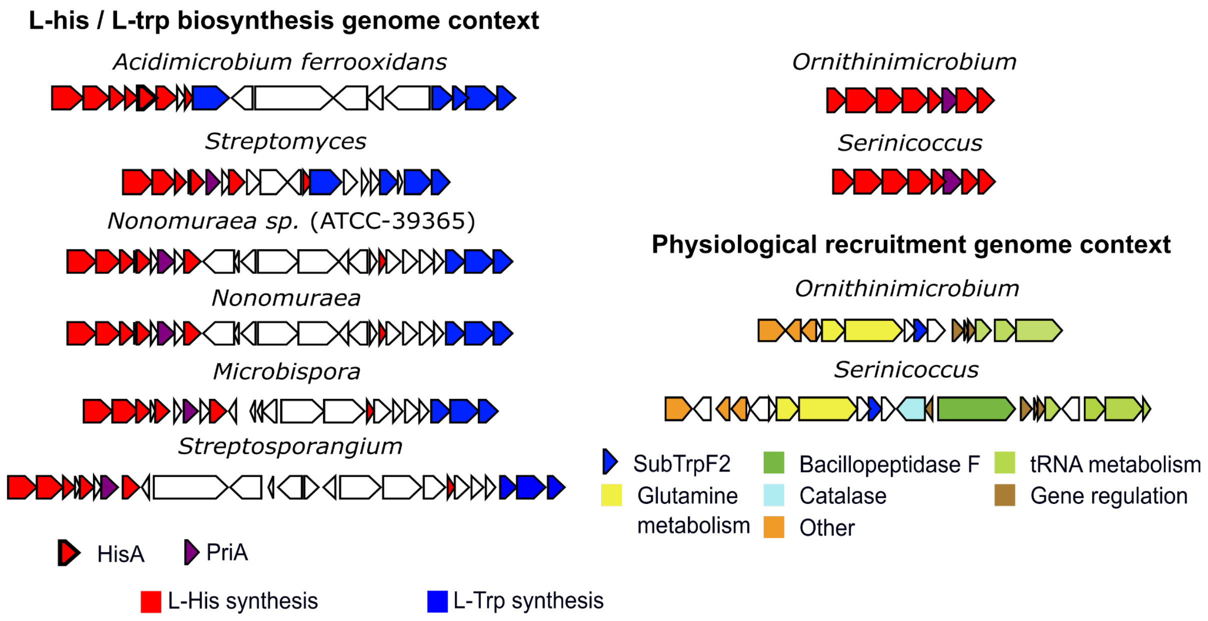

Figure S7. Distinct genomic contexts of HisA/PriA and SubTrpF2 homologs. Left: In *Acidimicrobium ferrooxidans* and *Streptosporangiaceae* genomes, HisA/PriA homologs are consistently recruited within amino acid biosynthetic operons. **Right:** In *Ornithinimicrobiaceae*, HisA and SubTrpF2 homologs co-occur but are embedded in distinct genomic contexts: HisA is associated with L-histidine biosynthesis, whereas SubTrpF2 is found in non–amino acid-related loci.

Table S3. Comparison of gene content of Adechlorin, Pentostatin, Coformacyn, and Pyrazomycin BGCs.

Adechlorin	Pentostatin	Coformacyn	Pyrazomycin	Function
adeE	-	-	-	Cation/H+ antiporter
adeD	-	-	-	MFS transporter
adeC	*penA*	-	*pyrS*	ATP phosphoribosyl-transferase
adeB	*penB*	*cofA*	*pyrP*	Short-chain dehydrogenase
adeA	*penC*	*cofB*	*pyrO*	SAICAR synthetase
adeF	-	*cof*	-	Aminotransferase
adeG	-	-	-	Dehydrogenase
adeH	-	-	-	ABC transporter, partial
adeI	-	-	-	ABC transporter
adeJ	-	-	-	Nudix hydrolase
adeK	-	-	-	Phosphoribosyl isomerase A
adeL	-	-	-	ATP phosphoribosyl-transferase
adeM	-	-	*pyrQ*	HAD family hydrolase
adeN	-	-	-	ABC transporter substrate-binding protein
adeO	-	-	-	Sugar ABC transporter permease
adeP	-	-	-	Nucleoside ABC transporter
adeQ	-	-	-	N,N-dimethylformamidase
adeR	-	-	-	Glucosamine-6-phosphate deaminase
adeS	-	-	-	Ribokinase
adeT	-	-	-	Bacterial regulatory protein, gntR family
adeU	-	-	-	Bacterial regulatory protein, gntR family
adeV	-	-	-	2OG-Fe(II) oxygenase
